# Cytotoxic effects of different mouthwash solutions on primary human articular chondrocytes and normal human articular cartilage – an *in vitro* study

**DOI:** 10.1007/s00784-023-05118-8

**Published:** 2023-06-17

**Authors:** Xiaoyu Cai, Jagadeesh K. Venkatesan, Gertrud Schmitt, Bashar Reda, Magali Cucchiarini, Matthias Hannig, Henning Madry

**Affiliations:** 1 Center of Experimental Orthopaedics, Saarland University, 66421 Homburg, Germany; 2 Department of Spine Surgery, First Affiliated Hospital of Xinjiang Medical University, Urumqi, China; 3 Clinic of Operative Dentistry, Periodontology and Preventive Dentistry, University Hospital, Saarland University, 66421 Homburg, Germany

**Keywords:** Articular chondrocyte, Articular cartilage, Human, Mouthwash, Octenidine dihydrochloride, Chlorhexidine gluconate, Toxicity

## Abstract

**Objectives:**

To compare the cytotoxicity of octenidine dihydrochloride and chlorhexidine gluconate at different concentrations on primary human articular chondrocytes and cartilage.

**Materials and methods:**

Primary cultures of human normal adult articular chondrocytes were exposed to octenidine dihydrochloride (0.001562%, 0.003125%, 0.00625%, 0.0125%, 0.025%, 0.05%, and 0.1%), chlorhexidine gluconate (0.003125%, 0.00625%, 0.0125%, 0.025%, 0.05%, 0.1%, and 0.2%), and control (Dulbecco’s modified Eagle medium or phosphate-buffered saline) for 30 s. Normal human articular cartilage explants were exposed to octenidine dihydrochloride (0.1% *versus* control) and chlorhexidine gluconate (0.1% *versus* control) for 30 s. The viability of human articular chondrocytes was measured by Trypan blue staining, Cell Proliferation Reagent WST-1, and Live/Dead staining. The proliferation of human chondrocytes was measured using the Cell Proliferation Reagent WST-1. The viability of human articular cartilage explants was measured by using Live/Dead staining.

**Results:**

Octenidine dihydrochloride and chlorhexidine gluconate exposure decreased cell viability and proliferation in a dose-dependent manner in primary human articular chondrocytes. Octenidine dihydrochloride and chlorhexidine gluconate exposure decreased cell viability in human articular cartilage explant cultures.

**Conclusion:**

The degree of toxicity varied between octenidine dihydrochloride and chlorhexidine gluconate, with chlorhexidine gluconate being less toxic than octenidine dihydrochloride at the same concentration. Additionally, both octenidine dihydrochloride and chlorhexidine gluconate evaluation had cytotoxic effects on human articular cartilage. Therefore, dosing for the antimicrobial mouthwash ingredients administration would ideally be determined to remain below IC50.

**Clinical relevance:**

These data support the *in vitro* safety of antimicrobial mouthwashes on primary adult human articular chondrocytes.

## Introduction

Antimicrobial mouthwashes are powerful mouthrinse formulations for infection control. Recent studies suggested their use also as a preventive measure against Coronavirus Disease 2019 (COVID-19), a respiratory disease induced by the severe acute respiratory syndrome coronavirus (SARS-CoV-2) [1] that has posed critical challenges for the public health, research, and medical communities [2–6]. This notion is based on the efficacy of antimicrobial mouthwashes to reduce the number of microorganisms in the oral cavity, prompting a reduction of microorganisms in aerosols [7]. This feature is particularly interesting as recent research indicates the relevance of aerosols in the spread of SARS-CoV-2 [8]. For example, Steinhauer et al. reported that an octenidine dihydrochloride-based mouthwash formulation was effective against SARS-CoV-2 within a contact time of only 15 s [6]. Moreover, several research groups have presented scientific evidence of a transient effect of antimicrobial mouthwashes in reducing SARS-CoV-2 viral load in saliva [9–11]. However, antimicrobial mouthwashes must be toxicologically harmless even for long-term therapy and should not affect nasal or oral mucous tissues or membranes, among which the nasal cartilage.

Chlorhexidine gluconate 0.2% is the gold standard antimicrobial mouthwash [12]. Nevertheless, in terms of safety, chlorhexidine gluconate displays cytotoxic effects on various cells including epithelial cells, fibroblasts, and stem cells [13–15]. Octenidine dihydrochloride shows ten times higher microbiostatic and microbiocidal effectiveness than chlorhexidine gluconate with better biocompatibility [16, 17] and may be considered a potent alternative to chlorhexidine gluconate [18]. Several lines of evidence suggest that the use of chlorhexidine gluconate and of octenidine dihydrochloride as mouthwash have cytotoxic effects on the cells of the oral cavity (e.g., gingival fibroblasts, periodontal ligament fibroblasts, and gingival epithelial cells) [14, 19, 20]. It is however crucial to evaluate the possible cytotoxic effects of antimicrobial mouthwashes on nasal chondrocytes to identify possible undesirable effects on the cartilage of the nasal septum that may be caused by contact, diffusion, active transport, or local blood flow. Yet, related studies on the effects of chlorhexidine gluconate- and octenidine dihydrochloride-based mouthwashes on nasal cartilaginous tissues are rare. Since articular chondrocytes have a comparable composition and structure to the nasal chondrocytes [21, 22], and nasal chondrocytes are also currently being evaluated as substitutes for knee articular chondrocytes in autologous cell-based therapies [23], they constitute a valuable alternative for such investigations.

The primary objective of this study was to determine the relative cytotoxicity of the antimicrobial mouthwashes octenidine dihydrochloride and chlorhexidine gluconate on human articular chondrocytes and human articular cartilage as an *in vitro* investigation of safety as possible candidates for preventive measures against Covid-19. Specifically, the objectives were to determine the half-maximal inhibitory concentration (IC50) which reflects the concentration of mouthwash ingredients at which 50% of the cells are viable and to compare IC50s between mouthwash ingredients as a preliminary screen to determine which mouthwash ingredients may be the safest. We employed primary cultures of isolated human adult articular chondrocytes and adult human articular cartilage explants to maximize clinical relevance. We hypothesized that the mouthwash ingredients evaluated would be cytotoxic to human chondrocytes and human cartilage in a dose-dependent manner. We also hypothesized that the degree of toxicity would vary between chlorhexidine gluconate and octenidine dihydrochloride, with chlorhexidine gluconate being less toxic than octenidine dihydrochloride at a similar concentration.

## Materials and methods

### Reagents

All reagents were purchased at Sigma (Munich, Germany) unless otherwise indicated. The Cell proliferation Reagent WST-1 was from Roche Applied Science (Mannheim, Germany). Trypan blue staining solution and the Live/Dead assay staining solution were obtained from Abcam (Cambridge, MA, USA). Chlorhexidine gluconate (ready-to-use solution 0.2%) and octenidine dihydrochloride (ready-to-use solution 0.1%) were from the Pharmacy of the Saarland University Hospital (Homburg, Germany). Plasticware was obtained from Falcon (Becton-Dickinson, Pont de Claix, France).

### Human articular cartilage explant culture

Human normal articular cartilage was retrieved from the unaffected parts of the knee subjected to total knee arthroplasty in osteoarthritis (OA) patients (*n* = 4, age 63–80, Mankin score 7–9) with previously informed consent [24]. This study has been approved by the Ethics Committee of the Saarland Physicians Council (*Ärztekammer des Saarlandes,* Approval Ha67/12). All protocols were in agreement with the Helsinki Declaration. By use of an aseptic technique, full-thickness cylindrical cartilage fragments were removed from the lateral and medial trochlear ridges of the distal aspect of the femur using a biopsy punch (diameter 6 mm, thickness 1 mm; Kai Europe, Solingen, Germany) in a standardized fashion. The articular cartilage explants were cultured in Dulbecco’s modified Eagle’s medium (DMEM) with 100 U/ml penicillin G, containing 10% fetal bovine serum (FBS) (growth medium) in a humidified atmosphere with 5% CO_2_ at 37 °C for 24 h prior to the addition of octenidine dihydrochloride and chlorhexidine gluconate.

### Primary human normal articular chondrocyte culture

Primary human normal articular chondrocytes (passage 1–2) were isolated from ~ 1 mm^3^ cartilage fragments retrieved from a 25-year-old patient undergoing unrelated cartilage surgery of the distal femur with previously informed consent following standard protocols [25, 26] and cultured in growth medium in 24-well plates at 20,000 cells/well for 24 h at 37 °C under 5% CO_2_ prior to the addition of octenidine dihydrochloride and chlorhexidine gluconate.

### Assessment of cell viability in cartilage explant cultures

Following the initial culture in a growth medium for 24 h, explants were washed with phosphate-buffered saline (PBS) and exposed to octenidine dihydrochloride (0.1% *versus* control) and chlorhexidine gluconate (0.1% *versus* control) for 30 s in 24-well plates (test volumes: always 200 µl). In order to maximize the clinical relevance, 1 ml growth medium was added directly to the cartilage explants in the presence of the antimicrobial mouthwashes without increasing the washing step after 30 s of exposure. The viability of human articular cartilage explants was measured 24 h after treatment by Live/Dead staining. According to the manufacturer’s instructions and visualization of fluorescence, staining was done using an Olympus CKX41 microscope using Olympus cellSens software (Olympus cellSens software; Olympus Life Sciences). Image J was used to count the live/dead pixels of each separated image [27].

### Assessment of cell proliferation and viability in monolayer culture

The effect of octenidine dihydrochloride with different concentrations (0.001562%, 0.003125%, 0.00625%, 0.0125%, 0.025%, 0.05%, and 0.1% *versus* control) and chlorhexidine gluconate with different concentrations (0.003125%, 0.00625%, 0.0125%, 0.025%, 0.05%, 0.1%, and 0.2% *versus* control) on primary adult human normal articular chondrocytes viability was assessed with a constant contact time of 30 s in 24-well plates (test volumes: always 200 µl). Control conditions included cell monolayers cultured in a growth medium with similar exposure to PBS instead of the antimicrobial mouthwashes. To maximize clinical relevance, no washing step was added after the 30 s of exposure and 1 ml growth medium was directly added to the cells in the presence of the antimicrobial mouthwashes. The proliferation of primary adult human normal articular chondrocyte monolayers was measured 24 h after treatment by using the Cell Proliferation Reagent WST-1 [25, 28]. Optical densities (ODs) at 450 nm were measured with a GENios spectrophotometer/fluorometer (Tecan, Mainz, Germany). The viability of primary adult human normal articular chondrocytes monolayers was measured 24 h after treatment by using the Trypan blue staining, Cell Proliferation Reagent WST-1, and Live/Dead staining, respectively. Percents of cell viabilities were determined as previously described [29].

### Statistical analysis

Data are expressed as mean ± standard deviation. Each test was performed 6 times. For monolayer cultures, IC50 was determined by normalizing dose-response for each concentration to control, transforming data to normalized dose-response *versus* log10 (concentration), and estimating IC50 by nonlinear regression in GraphPad Prism v8.4.1 (GraphPad Software Inc., San Diego, CA, USA) by fitting the data to a three-parameter sigmoid function (implemented as “log(inhibitor) *versus* dose-response.” In instances where the IC50 was outside the range of concentrations evaluated, or the data were not distributed in a sigmoid fashion following log transformation, the IC50 data were reported as a range of values as the exact value could not be determined based on the concentrations assessed. The t-test and the Mann-Whitney rank sum test were used where appropriate. *P* values less than 0.05 were considered statistically significant.

## Results

### Octenidine dihydrochloride exposure decreased cell viability and proliferation in a dose-dependent manner in primary adult human normal articular chondrocytes

**Fig. 1:**
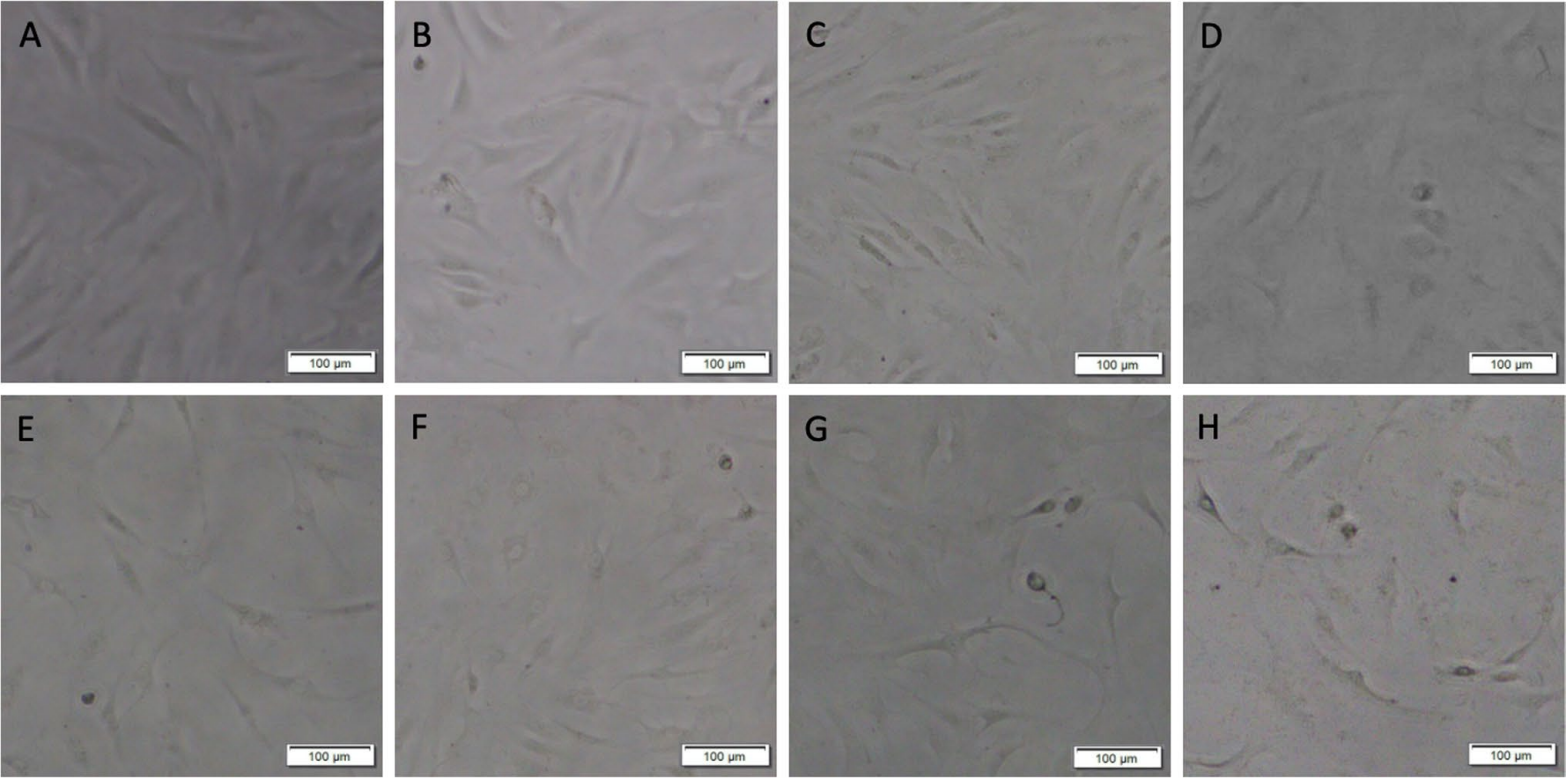
Effects of octenidine dihydrochloride on the morphology of primary adult human normal articular chondrocytes. Images of cultures left untreated (**A**) or treated with octenidine dihydrochloride at 0.001562% (**B**), 0.003125% (**C**), 0.00625% (**D**), 0.0125% (**E**), 0.025% (**F**), 0.05% (**G**), and 0.1% (**H**). Cells were viewed under a light microscope (scale bars: 100 μm). Untreated cells presented a characteristic spindle-like, fibroblastic morphology (**A**). Cells treated with octenidine dihydrochloride at 0.001562% and 0.003125% did not differ morphologically from the control cells (**B, C**). Cells treated with octenidine dihydrochloride at 0.00625%, 0.0125%, 0.025%, 0.05%, and 0.1% acquired a rounded, ruffled cell morphology (**D**–**H**)

**Fig. 2:**
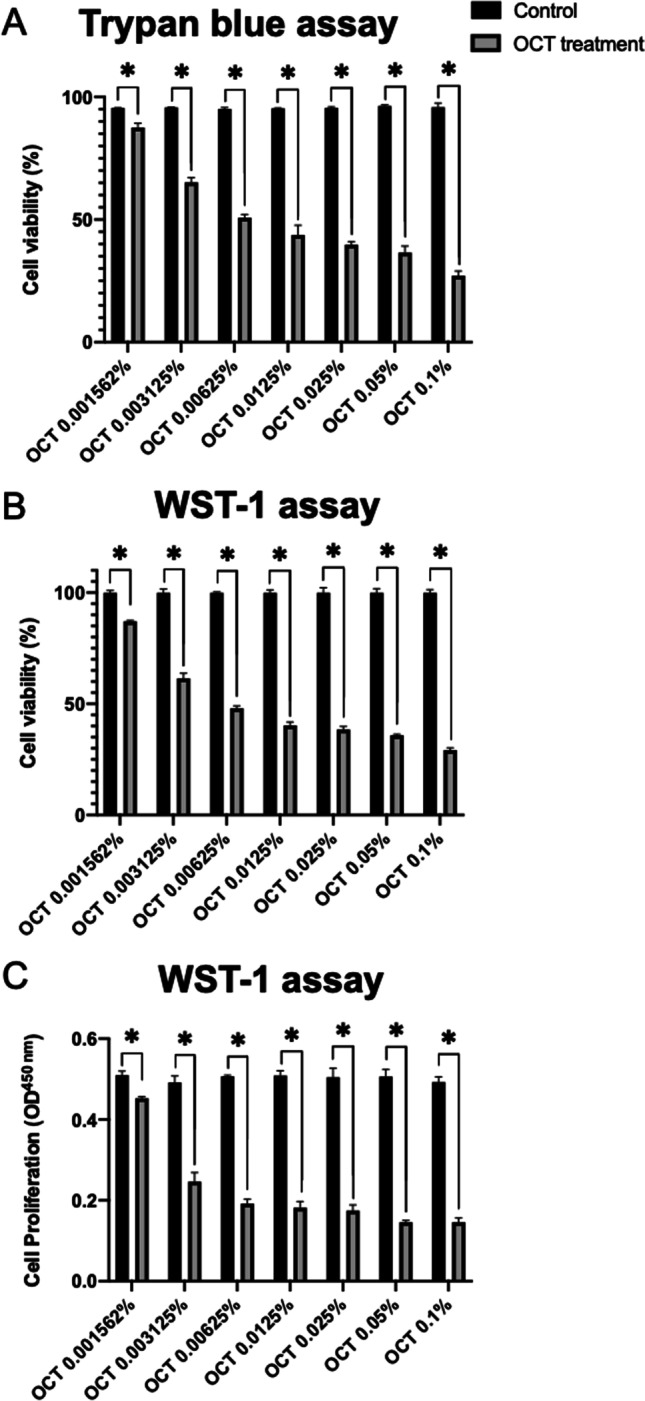
Effects of octenidine dihydrochloride on the viability and proliferation of primary adult human normal articular chondrocytes. Cell viability was monitored by trypan blue staining (**A**: % cell viability) and using the WST-1 assay (**B**: % cell viability; **C**: OD^450 nm^) after treatment with octenidine dihydrochloride at 0.001562%, 0.003125%, 0.00625%, 0.0125%, 0.025%, 0.05%, and 0.1%. OCT, octenidine dihydrochloride. *Statistically significant *versus* control group

**Fig. 3:**
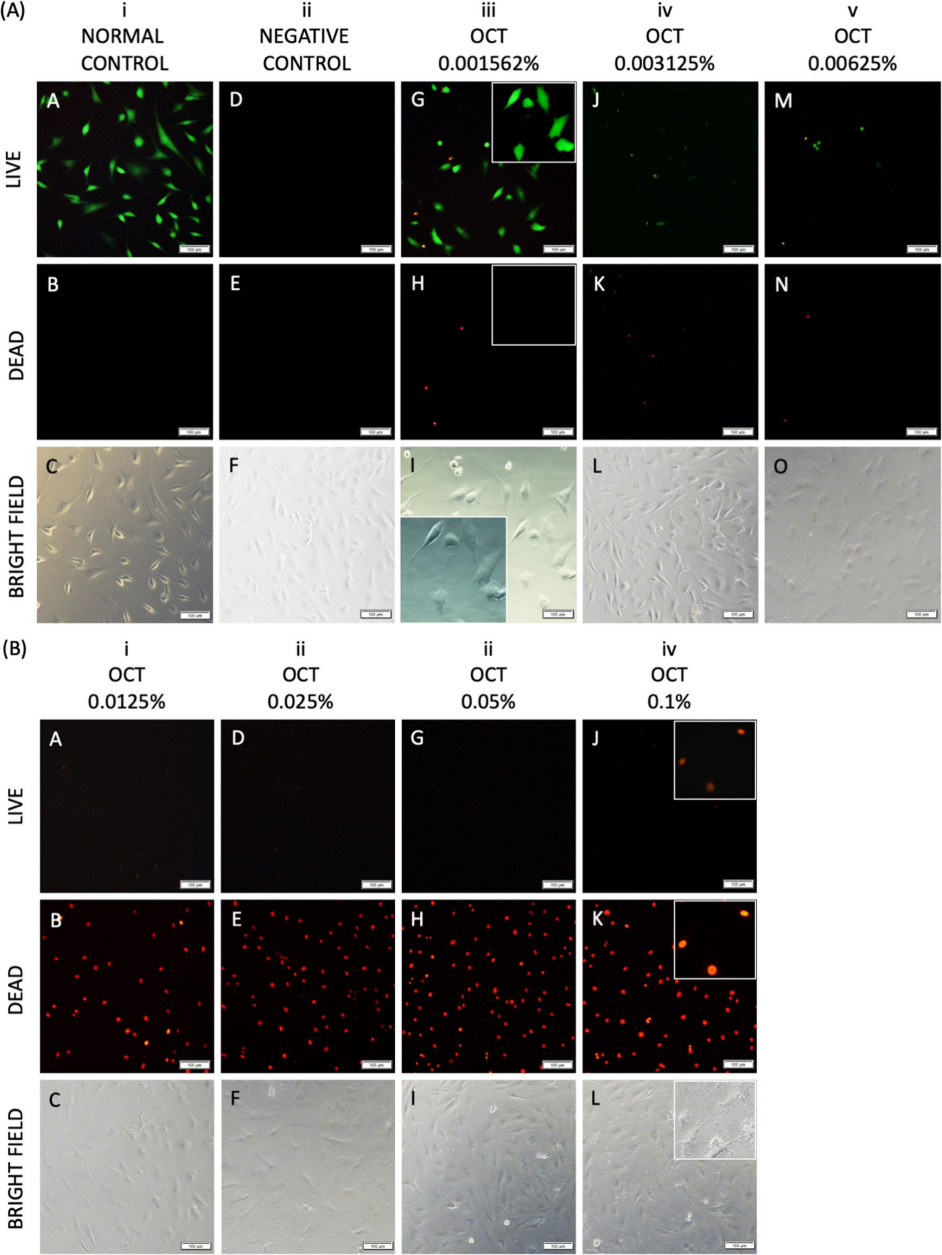
Effects of octenidine dihydrochloride on the viability of primary adult human normal articular chondrocytes, as analyzed by Live/Dead fluorescence staining. **A**, (**A**–**C**) Normal control. (**D**–**F**) Negative control. (**A, D, G, J, M**) Images of live cells (green fluorescence). (**B, E, H, K, N**) Images of dead cells (red fluorescence). (**C, F, I, L, O**) Images of cells under a light microscope. **B**, (**A, D, G, J**) Images of live cells. (**B, E, H, K**) Images of dead cells. (**C, F, I, L**) Images of cells under a light microscope. Scale bars: 100 μm

Exposure to octenidine dihydrochloride for 30 s resulted in macroscopic morphological changes, decreased viability, and proliferation of primary adult human normal articular chondrocytes in a dose-dependent manner when octenidine dihydrochloride concentration was titrated from 0.003125 to 0.1% (Figs. 1, 2, and 3). Primary adult human normal articular chondrocytes did not differ morphologically from control cells when treated with octenidine dihydrochloride at 0.001562% and 0.003125%. Cells acquired a rounded, ruffled cell morphology at the higher doses of 0.00625%, 0.0125%, 0.025%, 0.05%, and 0.1% octenidine dihydrochloride (Fig. 1).

The cell viability monitored by trypan blue assay staining after treatment with octenidine dihydrochloride at 0.001562%, 0.003125%, 0.00625%, 0.0125%, 0.025%, 0.05%, and 0.1% showed the results as 87.5 ± 1.8%, 65.2 ± 1.9%, 50.8 ± 1.3%, 43.7 ± 4%, 39.8 ± 1.2%, 36.5 ± 2.7%, and 27.1 ± 1.9%, respectively. The cell viability of octenidine dihydrochloride treatment groups was significantly lower than the control groups to all concentrations starting from 0.001562% (*P* < 0.001, respectively) (Fig. 2). Cell viability as monitored by the WST-1 assay after identical treatment with graded concentrations of octenidine dihydrochloride was 87.1 ± 0.4%, 61.5 ± 2.3%, 48.0 ± 1.2%, 40.33 ± 1.6%, 38.4 ± 1.4%, 36.0 ± 0.4%, and 29.2 ± 1.0%, respectively. The cell viability of octenidine dihydrochloride treatment groups was significantly lower than the control groups to all concentrations starting from 0.001562% (*P* < 0.001, respectively) (Fig. 2).

Analysis of cell proliferation revealed decreased proliferative indices upon treatment with octenidine dihydrochloride at 0.001562%, 0.003125%, 0.00625%, 0.0125%, 0.025%, 0.05%, and 0.1% (up to 3.4-fold decrease; *P* < 0.001 at all concentrations compared with the control group) (Fig. 2). The half-maximal inhibitory concentration (IC50), reflecting the dose of octenidine dihydrochloride at which approximately 50% of the cells were alive, was 0.01047% (Table 1).

**Table 1:** Half-maximal inhibitory concentration (IC50) values of octenidine dihydrochloride and chlorhexidine gluconate

	Octenidine dihydrochloride	Chlorhexidine gluconate
IC50	0.01047%	0.06014%

### Chlorhexidine gluconate exposure decreased cell viability and proliferation in a dose-dependent manner in primary adult human normal articular chondrocytes

**Fig. 4:**
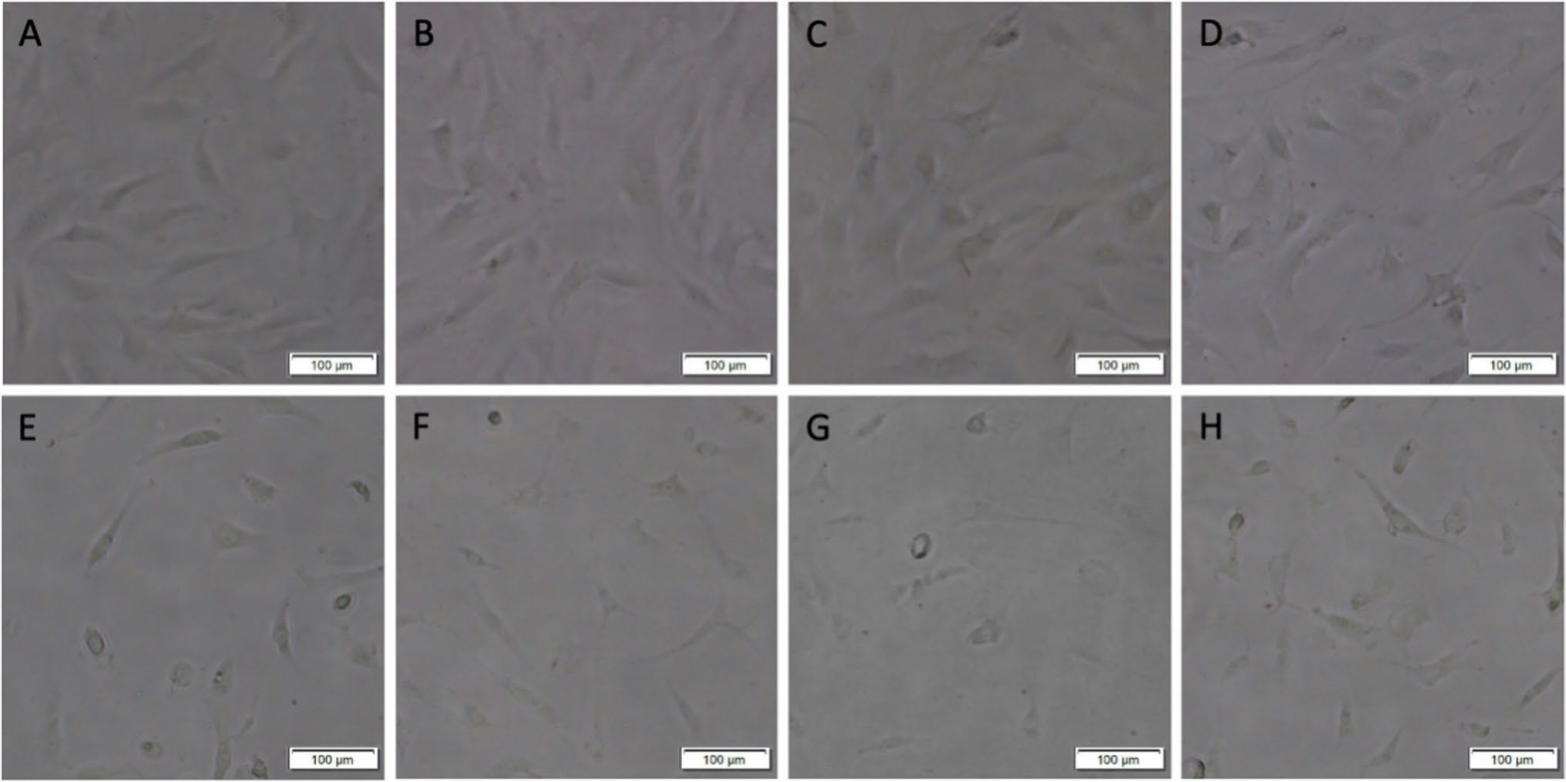
Effects of chlorhexidine gluconate on the morphology of primary adult human normal articular chondrocytes. Images of cultures left untreated (**A**) or treated with chlorhexidine gluconate at 0.003125% (**B**), 0.00625% (**C**), 0.0125% (**D**), 0.025% (**E**), 0.05% (**F**), 0.1% (**G**), and 0.2% (**H**). Cells were viewed under a light microscope (scale bars: 100 μm). Untreated cells presented a characteristic spindle-like fibroblastic morphology (**A**). Cells treated with chlorhexidine gluconate at 0.003125%, 0.00625%, and 0.0125% did not differ morphologically from the control cells (**B**–**D**). Cells treated with chlorhexidine gluconate at 0.025%, 0.05%, 0.1%, and 0.2% acquired a rounded, ruffled cell morphology (**E**–**H**)

**Fig. 5:**
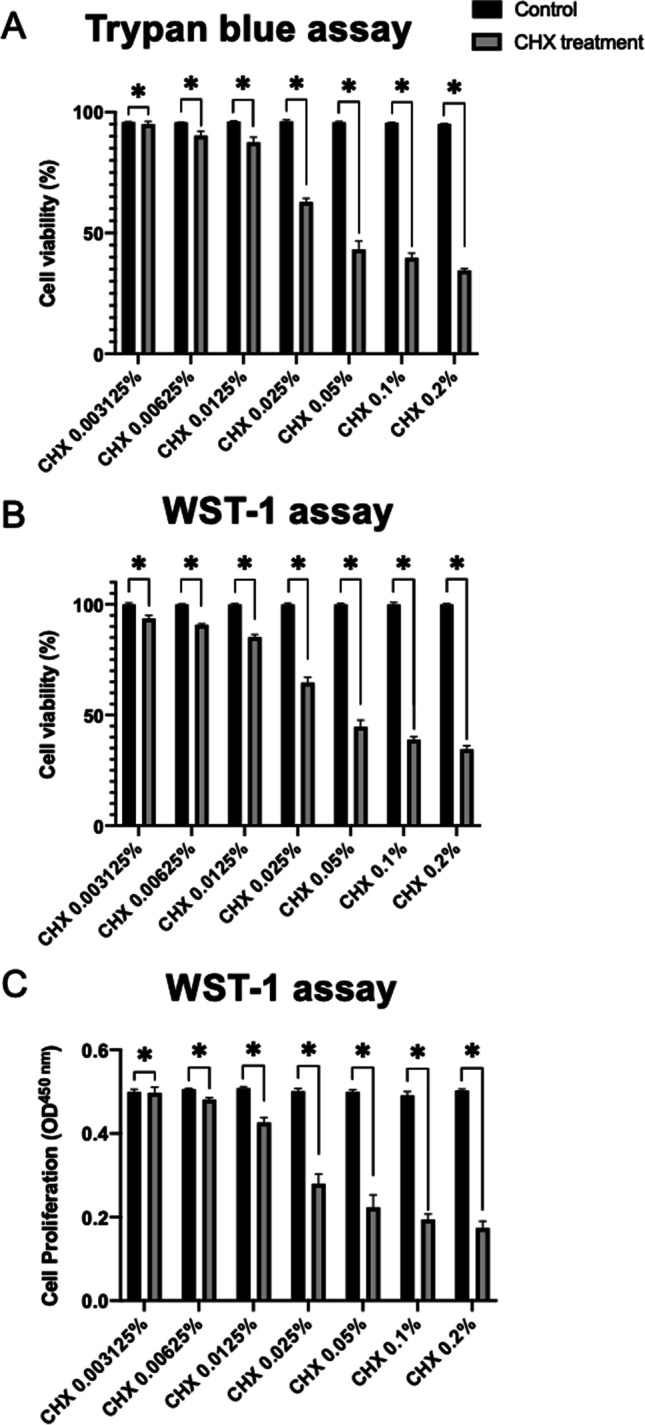
Effects of chlorhexidine gluconate on the viability and proliferation of primary adult human normal articular chondrocytes. Cell viability was monitored by trypan blue staining (**A**: % cell viability) and using the WST-1 assay (**B**: % cell viability; **C**: OD^450 nm^) after treatment with chlorhexidine gluconate at 0.003125%, 0.00625%, 0.0125%, 0.025%, 0.05%, 0.1%, and 0.2%. CHX, chlorhexidine gluconate. *Statistically significant *versus* control group

**Fig. 6:**
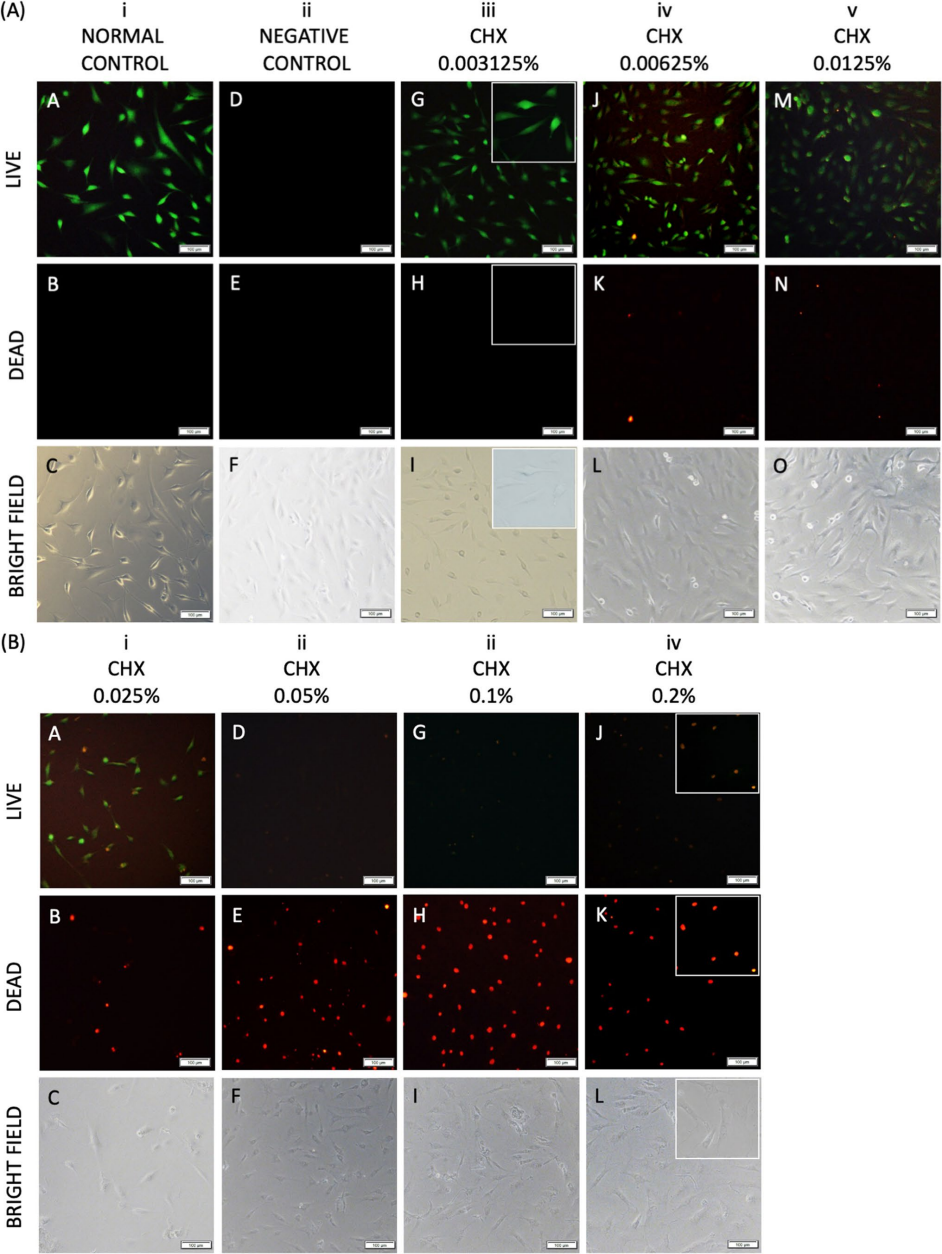
Effects of chlorhexidine gluconate on the viability of primary adult human normal articular chondrocytes, as analyzed by Live/Dead fluorescence staining. **A**, (**A**–**C**) Normal control. (**D**–**F**) Negative control. (**A, D, G, J, M**) Images of live cells (green fluorescence). (**B, E, H, K, N**) Images of dead cells (red fluorescence). (**C, F, I, L, O**) Images of cells under a light microscope. **B**, (**A, D, G, J**) Images of live cells. (**B, E, H, K**) Images of dead cells. (**C, F, I, L**) Images of cells under a light microscope. Scale bars: 100 μm

Identical exposure to chlorhexidine gluconate for 30 s resulted in macroscopic morphological changes, decreased viability and proliferation of primary adult human normal articular chondrocytes in a dose-dependent manner when chlorhexidine gluconate concentration was titrated from 0.003125 to 0.1% (Figs. 4, 5, and 6). Primary adult human normal articular chondrocytes did not differ morphologically from control cells when treated with chlorhexidine gluconate at 0.003125%, 0.00625%, and 0.0125%. However, cells acquired a rounded, ruffled cell morphology at the higher doses of 0.025%, 0.05%, 0.1%, and 0.2% chlorhexidine gluconate (Fig. 4).

The cell viability monitored by trypan blue assay staining after treatment with chlorhexidine gluconate at 0.003125%, 0.00625%, 0.0125%, 0.025%, 0.05%, 0.1%, and 0.2% showed the results as 95.1 ± 1.1%, 90.3 ± 1.8%, 87.5 ± 2.1%, 62.9 ± 1.5%, 43.2 ± 3.4%, 39.7 ± 1.9%, and 34.5 ± 0.8%, respectively. The cell viability of chlorhexidine gluconate treatment groups was significantly lower than the control groups to all concentrations starting from 0.00625% (*P* < 0.001, respectively) (Fig. 5). There was no significant difference between the chlorhexidine gluconate treatment group at 0.003125% and the control group (*P* > 0.09) (Fig. 5). Cell viability as monitored by WST-1 assay after identical treatment with graded concentrations of chlorhexidine gluconate was 93.64 ± 1.3%, 90.8 ± 0.5%, 85.2 ± 1.1%, 64.7 ± 2.3%, 44.7 ± 3.0%, 38.9 ± 1.3%, and 34.5 ± 1.7%, respectively. The cell viability of chlorhexidine gluconate treatment groups was significantly lower than the control groups to all concentrations starting from 0.003125% (*P* < 0.001, respectively) (Fig. 5).

Analysis of cell proliferation revealed decreased proliferative indices upon treatment with chlorhexidine gluconate at 0.00625%, 0.0125%, 0.025%, 0.05%, 0.1%, and 0.2% (up to 2.9-fold decrease; *P* < 0.001 at all concentrations compared with the control group) (Fig. 5). There was no significant difference between the chlorhexidine gluconate treatment group at 0.003125% and the control group (*P* > 0.06) (Fig. 5). The half-maximal inhibitory concentration (IC50) of chlorhexidine gluconate was 0.06014% (Table 1).

### Octenidine dihydrochloride and chlorhexidine gluconate exposure decreased cell viability in human articular cartilage explant cultures

**Fig. 7:**
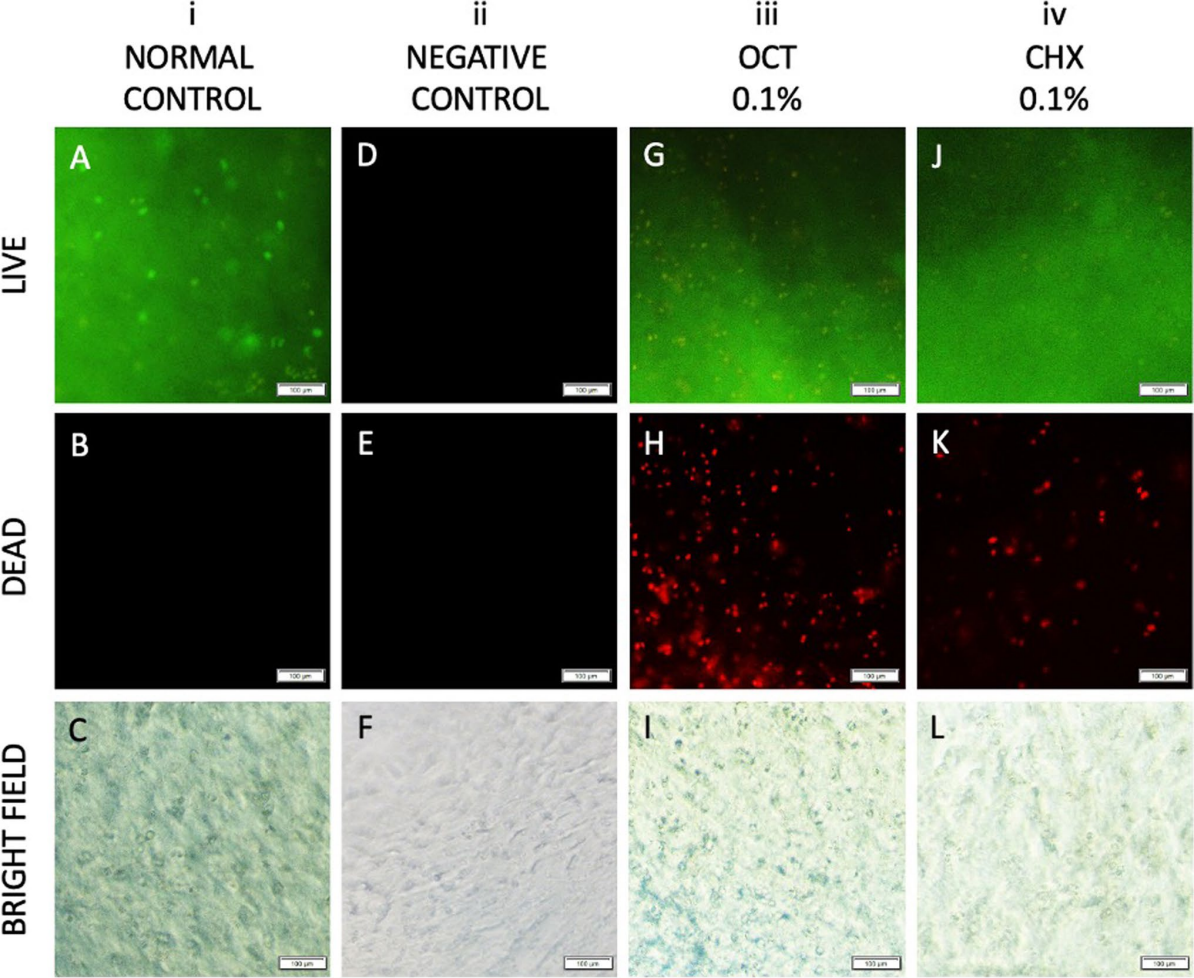
Effects of octenidine dihydrochloride and chlorhexidine gluconate on the viability of adult human articular cartilage explant tissues, as analyzed by Live/Dead fluorescence staining. (**A**–**C**) Normal control. (**D**–**F**) Negative control. (**A, D, G, J**) Images of live cells (green fluorescence). (**B, E, H, K**) Images of dead cells (red fluorescence). (**C, F, I, L**) Images of cells under a light microscope. Scale bars: 100 μm

Exposure of human articular cartilage explants to 0.1% octenidine dihydrochloride for 30 s resulted in a considerably decreased viability of adult human articular chondrocytes within the human cartilage cultures compared with control explant cultures (Fig. 7). Likewise, exposure to 0.1% chlorhexidine gluconate for 30 s resulted in decreased viability of adult human articular chondrocytes within human articular cartilage explant cultures compared with control explant cultures (Fig. 7).

## Discussion

The present data provide important comparative *in vitro* information on the safety of two major antimicrobial mouthwashes, octenidine dihydrochloride and chlorhexidine gluconate on primary human normal articular chondrocytes and human normal articular cartilage. The major finding is that chlorhexidine gluconate is less toxic than octenidine dihydrochloride at the same concentrations in primary monolayer cultures of adult human normal articular chondrocytes. Octenidine dihydrochloride and chlorhexidine gluconate evaluation showed cytotoxicity to primary adult human normal articular chondrocytes in a dose-dependent manner. Moreover, chlorhexidine gluconate at 0.1% is less toxic than octenidine dihydrochloride at 0.1% in explant cultures of human articular cartilage. Additionally, the IC50 of octenidine dihydrochloride is lower than that of octenidine dihydrochloride. This study provides a platform for further investigations on the *in vivo* efficacy of antimicrobial mouthwashes.

Cytotoxic effects of octenidine dihydrochloride and chlorhexidine gluconate on human cells (e.g., gingival fibroblasts, nasal epithelial cells, myoblasts, osteoblasts, and stem cells) have been investigated in many other studies to compare the toxicity of these compounds [13–15, 30, 31]. Garbrecht et al. exposed individual human osteochondral explant plugs to chlorhexidine (0.01% and 0.5%); 0.5% chlorhexidine showed significant cytotoxicity, with viability reduced to less than 40% by day 6 [32]. Chondrocytes exposed to 0.01% chlorhexidine maintained viability. Campbell et al. showed in the context of a strategy of salvaging contaminated osteochondral allografts that a pulse lavage with 0.002% chlorhexidine gluconate does not cause significant cell death within 7 days after exposure, while chlorhexidine gluconate at concentrations > 0.002% significantly decreases human articular chondrocyte viability within 1 to 2 days after exposure [33]. Schmidt and colleagues reported that octenidine dihydrochloride could be recommended as an alternative to chlorhexidine gluconate because of its lower cytotoxic potential [30]. However, the concentration of chlorhexidine gluconate in this study was 0.2% [30], and no data were provided about the concentration of octenidine dihydrochloride. Eick et al. demonstrated that commercially available chlorhexidine gluconate mouthwash has a very strong cytotoxic effect on the gingival fibroblasts in the MTT assay at different concentrations (0.01%, 0.06%, 1%, and 2%) with a contact time of 1 min [14]. Here, the concentrations of both octenidine dihydrochloride and chlorhexidine gluconate were titrated from 0.003125 to 0.1%, and the contact time was 30 s. Our results indicated that chlorhexidine gluconate is less toxic than octenidine dihydrochloride in the same concentration as 0.003125%, 0.00625%, 0.0125%, 0.025%, 0.05%, and 0.1% on primary adult human normal articular chondrocytes. The data presented the cell viability tested by trypan blue assay was in good agreement with WST-1 assay.

The cytotoxicity observed following octenidine dihydrochloride and chlorhexidine gluconate exposure in monolayer cell culture and explant tissues was consistent. Our results indicate that chlorhexidine gluconate is less toxic than octenidine dihydrochloride in the same concentration as 0.1% on human cartilage explant tissues. Cell death was evident within the cartilage explant at both 0.1% octenidine dihydrochloride and 0.1% chlorhexidine gluconate compared to the controls. Because 0.1% of octenidine dihydrochloride is the most commonly used mouthwash ingredient [34], the concentration of 0.1% was chosen here. An investigation of the effects of octenidine dihydrochloride and chlorhexidine gluconate on chondrocytes and cartilage explant tissue viability *in vivo* is warranted to extend these data.

Human gingival fibroblasts [14], human gingival cells [35], human periodontal ligament fibroblasts [19], human gingival epithelial cells [20], and odontoblast-like cells [36] have been tested *in vitro* to elucidate the cytotoxic activity of chlorhexidine gluconate. For octenidine dihydrochloride, human gingival fibroblasts and nasal epithelial cells [30] have been tested *in vitro* to assess their cytotoxic activity. Our study used primary human normal articular chondrocytes because nasal chondrocytes have a comparable composition and structure as articular chondrocytes [21, 22]. Nasal chondrocytes are currently being evaluated as substitutes for knee articular chondrocytes in autologous cell-based therapies [23].

The IC50 is a measure of the effectiveness of a compound in inhibiting biological or biochemical function. This quantitative measure indicates how much of a particular drug or other substance is needed to inhibit a given biological process by half. According to the Food and Drug Administration, IC50 represents the concentration of a drug that is required for 50% inhibition *in vitro*. As shown in Table 1, the IC50 of octenidine dihydrochloride was 0.01047%, and the value of IC50 of chlorhexidine gluconate was 0.06014%. Thus, the results are in good agreement with that of the WST-1 assay. Furthermore, the IC50 values have implications for further research on the cytotoxic effects of octenidine dihydrochloride and chlorhexidine gluconate *in vivo*.

Limitations of this study include the use of human articular cartilage and the lack of *in vivo* evaluation on the potential protective effect of the complex supra-molecular salivary film of the mucosal pellicle, together with the nasal epithelium, and the fact that just a small aerosolized part of mouthwash reaches the nose [37]. Although the findings have to be interpreted with caution, the strengths of this study are the comprehensive examination of a wide range of dose-dependent effects including IC50 values, the short (30 s) contact time matching the clinical mouthwash’s utilization instructions, and the use of human primary chondrocyte and explant cultures where the cells remained in their natural three-dimensional environment.

## Conclusions

Octenidine dihydrochloride and chlorhexidine gluconate decrease the viability and proliferation of primary adult human normal articular chondrocytes in a dose-dependent manner. The degree of toxicity varied between octenidine dihydrochloride and chlorhexidine gluconate, with chlorhexidine gluconate being less toxic than octenidine dihydrochloride at the same concentration. Additionally, both octenidine dihydrochloride and chlorhexidine gluconate were cytotoxic to human normal articular cartilage. Dosing antimicrobial mouthwash ingredients administration would ideally be determined to remain below IC50.

## Data Availability

All the data obtained and/or analyzed associated with the current study were available from the corresponding authors upon reasonable request.
